# UM171A-induced ROS promote antigen cross-presentation of immunogenic peptides by bone marrow-derived mesenchymal stromal cells

**DOI:** 10.1186/s13287-021-02693-z

**Published:** 2022-01-10

**Authors:** Natasha Salame, Jean-Pierre Bikorimana, Nehme El-Hachem, Wael Saad, Mazen Kurdi, Jing Zhao, Nicoletta Eliopoulos, Riam Shammaa, Moutih Rafei

**Affiliations:** 1 Department of Biomedical Sciences, Université de Montréal, Montreal, QC Canada; 2 Department of Microbiology, Infectious Diseases and Immunology, Université de Montréal, Montreal, QC Canada; 3 Department of Pharmacology and Physiology, Université de Montréal, Montreal, QC Canada; 4 Pediatric Hematology-Oncology Division, Centre Hospitalier Universitaire Sainte-Justine Research Centre, Montreal, QC Canada; 5 Laboratory of Experimental and Clinical Pharmacology, Department of Chemistry and Biochemistry, Faculty of Sciences, Lebanese University, Hadat, Lebanon; 6 Lady Davis Institute for Medical Research, Jewish General Hospital, Montreal, QC Canada; 7 Department of Surgery, McGill University, Montreal, QC Canada; 8 Department of Family and Community Medicine, University of Toronto, Toronto, ON Canada; 9 Canadian Centers for Regenerative Therapy, Toronto, ON Canada; 10 IntelliStem Technologies Inc., Toronto, ON Canada; 11 Molecular Biology Program, Université de Montréal, Montreal, QC Canada; 12 Department of Microbiology and Immunology, McGill University, Montreal, QC Canada

**Keywords:** Mesenchymal stromal cells, Antigen cross-presentation, UM171a, Reactive oxygen species, PSMB8, Electron transport chain, Anti-oxidants, Cellular vaccine, Anti-tumoral immunity

## Abstract

**Background:**

Mesenchymal stromal cells (MSCs) have been extensively used in the clinic due to their exquisite tissue repair capacity. However, they also hold promise in the field of cellular vaccination as they can behave as conditional antigen presenting cells in response to interferon (IFN)-gamma treatment under a specific treatment regimen. This suggests that the immune function of MSCs can be pharmacologically modulated. Given the capacity of the agonist pyrimido-indole derivative UM171a to trigger the expression of various antigen presentation-related genes in human hematopoietic progenitor cells, we explored the potential use of UM171a as a means to pharmacologically instill and/or promote antigen presentation by MSCs.

**Methods:**

Besides completing a series of flow-cytometry-based phenotypic analyses, several functional antigen presentation assays were conducted using the SIINFEKL-specific T-cell clone B3Z. Anti-oxidants and electron transport chain inhibitors were also used to decipher UM171a’s mode of action in MSCs. Finally, the potency of UM171a-treated MSCs was evaluated in the context of therapeutic vaccination using immunocompetent C57BL/6 mice with pre-established syngeneic EG.7T-cell lymphoma.

**Results:**

Treatment of MSCs with UM171a triggered potent increase in H2-K^b^ cell surface levels along with the acquisition of antigen cross-presentation abilities. Mechanistically, such effects occurred in response to UM171a-mediated production of mitochondrial-derived reactive oxygen species as their neutralization using anti-oxidants or Antimycin-A mitigated MSCs’ ability to cross-present antigens. Processing and presentation of the immunogenic ovalbumin-derived SIINFEKL peptide was caused by de novo expression of the *Psmb8* gene in response to UM171a-triggered oxidative stress. When evaluated for their anti-tumoral properties in the context of therapeutic vaccination, UM171a-treated MSC administration to immunocompetent mice with pre-established T-cell lymphoma controlled tumor growth resulting in 40% survival without the need of additional supportive therapy and/or standard-of-care.

**Conclusions:**

Altogether, our findings reveal a new immune-related function for UM171a and clearly allude to a direct link between UM171a-mediated ROS induction and antigen cross-presentation by MSCs. The fact that UM171a treatment modulates MSCs to become antigen-presenting cells without the use of IFN-gamma opens-up a new line of investigation to search for additional agents capable of converting immune-suppressive MSCs to a cellular tool easily adaptable to vaccination.

**Graphical abstract:**

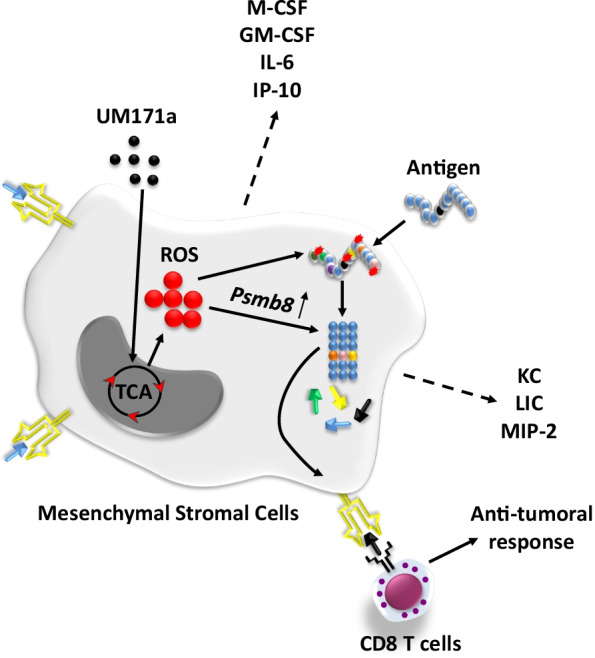

**Supplementary Information:**

The online version contains supplementary material available at 10.1186/s13287-021-02693-z.

## Background

Several characteristics support the extensive use of culture-adapted mesenchymal stromal cells (MSCs) as cellular biopharmaceuticals [1, 2]. These include: (1) simple isolation protocols from small volumes of bone marrow (BM) aspirates, (2) ease of in vitro proliferation/expansion, (3) standard and defined culture medium, (4) low senescence through multiple passages, (5) ability to be gene modified, and (6) distinct in vivo migration capabilities toward damaged or inflamed tissues [1–7]. The latter point combined with the mesenchymal differentiation plasticity of MSCs explain why these cells are extensively used for tissue repair and wound healing [1–7]. Besides, MSCs can display remarkable immunomodulatory properties [1–7]. These immune functions are, however, influenced by the surrounding pro-inflammatory environment [8]. For instance, MSC stimulation with low interferon (IFN)-gamma doses (< 25 pg/ml) triggers antigen-presenting cell (APC)-like functions, whereas higher and/or sustained IFN-gamma concentrations correlate with MSCs switching roles to immune-suppressor cells [8]. Physiologically, this means that MSCs could act as “gatekeepers” in the BM to preserve hematopoietic homeostasis during exacerbated inflammation [8]. From a therapeutic point of view, however, high or sustained exposure of MSCs to IFN-gamma may not be suitable for cellular vaccination as it can halt their APC-like function 12 h post-priming [6, 8–11]. Besides, IFN-gamma-primed MSCs express the immune checkpoint inhibitor PD-L1, which is known to impair metabolic and effector function of cytotoxic T lymphocytes [6, 8–11]. Furthermore, IFN-gamma treatment of human MSCs did not promote antigen presentation. Instead, it enhanced their capacity to suppress T-cell proliferation and graft-versus-host disease progression in humanized mice via production of indoleamine 2,3-dioxygenase [12]. Similar outcomes were observed when responding T cells were co-cultured with antigen-pulsed IFN-gamma-stimulated human MSCs [13]. Thus, the design of novel pharmacological strategies capable of triggering consistent pro-inflammatory functions in both murine and human MSCs while bypassing the above-mentioned limitations remains a central goal for the development of immune-stimulatory MSC-based therapeutics.

Several stem cell "pharmaco-optimization" strategies were previously reported to enhance MSCs’ innate function as a means to ensure the development of a desirable therapeutic effect. For instance, in vitro pre-conditioning of MSCs with the anti-oxidant hormone melatonin was shown to improve their survival and anti-apoptosis effects [14]. Likewise, pre-conditioning of MSCs or other stem cell products with Celastrol, a natural compound known to promote anti-oxidant responses through activation of the nuclear factor erythroid 2-related factor 2 gene, represents another example showing how pharmacological stimulation enhances the endogenous protective effects of MSCs by increasing cell viability and therapeutic efficiency [15, 16]. Although these examples demonstrate that it is indeed feasible to pharmacologically modulate MSC function, no drug/compound besides IFN-gamma was ever reported to trigger APC-like properties in MSCs.

We show in this study how culture-adapted MSCs treated with the pyrimido-indole derivative UM171a exhibit enhanced production of mitochondrial-derived superoxide anion. As a result, treated MSCs up-regulate their major histocompatibility complex (MHC) I expression and acquire the capacity to cross-present immunogenic peptides from captured soluble protein. When tested as a therapeutic cellular vaccine, antigen-pulsed UM171a-treated MSCs significantly interfered with the growth of pre-established solid T-cell lymphoma. Altogether, these studies indicate that UM171a-treated MSCs can indeed serve as a possible alternative to standard dendritic cells in the future design of cancer vaccines.

## Methods

### Animals and ethics

All C57BL/6 female mice (6- to 8-week-old) used in this study were purchased from Jackson Laboratories (Bar Harbor, ME, USA) and housed in a pathogen-free environment at the animal facility of the Institute for Research in Immunology and Cancer (IRIC). Animal protocols were approved by the Animal Care Committee of Université de Montréal.

### Cell lines, primary cells, and reagents

The EG.7 and B3Z cell lines were kindly provided by Dr. Jacques Galipeau (University of Wisconsin-Madison, WI, USA). Murine embryonic fibroblasts (MEFs) were kindly provided by Dr. John Stagg (CR-CHUM, QC, Canada). Human umbilical cord (UC)-derived MSCs and their specific culture media were purchased from RoosterBio Inc. (Frederick, MD, USA). All remaining cell culture media and reagents were purchased from Wisent Bioproducts (Saint-Jean-Baptiste, QC, CANADA). The anti-endothelial protein C receptor (EPCR) antibody, the anti-PD-L1 neutralizing antibody, and the IFN-gamma/interleukin (IL)-2 Quantikine ELISAs were purchased from R&D Systems (Minneapolis, MN, USA). All remaining antibodies used in flow-cytometry were purchased from BD Biosciences (San Jose, CA, USA). The murine indoleamine 2,3-dioxygenase (IDO-1)-specific ELISA was purchased from Cusabio (Houstan, TX, USA). The albumin from chicken egg white (OVA), Accutase®, Rotenone, Malonate, Antimycin A, Sodium Azide, Oligomycin, MitoTEMPO, *α*-tocopherol, *N*-acetylcysteine (NAC), lipopolysaccharide (LPS), and Chlorophenol red-*β*-d-galactopyranoside (CPRG) were purchased from Sigma-Aldrich (St-Louis, MI, USA). The SIINFEKL peptide was synthesized by GenScript (NJ, USA). Recombinant IFN-gamma and granulocyte macrophage-colony stimulating factor (GM-CSF) were purchased from Peprotech (Rocky Hill, NJ, USA). The UM171a compound was provided by ExCellThera (Montreal, QC, CANADA). Alexa Fluor® 647-conjugated OVA, OVA-DQ®, and MitoSox™ were purchased from ThermoFisher (Waltham, MA, USA). The CD8 T-cell isolation kit was purchased from STEMCELL Technologies (Vancouver, BC, CANADA). Amicon Ultra-4 centrifugal filters were purchased from Millipore-Sigma (Burlington, MA, USA).

### Generation of primary BM-derived MSCs

To generate mouse primary MSCs, BM was flushed from femurs of a female C57BL/6 mouse and cultured in alpha-MEM supplemented with 10% fetal bovine serum (FBS), 50 U/mL Penicillin and 50 μg/ml Streptomycin. The media was changed every 2–3 days until MSC colonies were apparent. Following 2–3 passages, MSCs were phenotyped by flow-cytometry using antibodies against CD44, CD45, CD73, CD90.1, CD105, and H2-K^b^ diluted according to manufacturer’s instructions. After washing using the staining buffer, cells were re-suspended in 400 μl of staining buffer. The samples were acquired by BD FACS Diva on CANTOII and then, analyzed using FlowJoV10.

### Assessment of the UM171a maximum tolerated dose (MTD)

To identify the MSC MTD for UM171a, 5 × 10^4^ plated MSCs were treated with ascending doses of the compound (35 to 8000 nM) for 72 h. Treated cells were then washed, detached and counted using trypan blue to differentiate between live and dead cells. The highest dose with no toxicity or decreased proliferation effects was selected for subsequent studies.

### Antigen cross-presentation assay

To assess antigen cross-presentation, MSCs or MEFs were first seeded in a 24-well plate at 1.5 × 10^4^ cells per well. The following day, adherent cells were treated with 35, 250 or 1000 nM of UM171a or equivalent DMSO concentration for three days prior to pulsing with OVA at 5 mg/ml for 5–6 h. A similar approach was used for the SIINFEKL peptide (at 0.1 μg/ml) except that the pulsing period was 2–3 h. Once the pulsing was completed, cells were washed twice to remove excess antigen/peptide followed by the addition of 5 × 10^5^ B3Z (SIINFEKL/H2-K^b^-specific T-cell line) for 15–17 h. The following day, all cells were lysed and then, stained with a CPRG solution for 18 h at 37 °C. The optical density signal was detected using a SynergyH1 microplate reader (Biotek, Winooski, VT, United States). For all experiments using inhibitors or antioxidants, the same assay was conducted and inhibitors/antioxidants were added as detailed elsewhere. A similar setup was used for OT-I-based antigen presentation assays except that isolated OT-I-derived CD8 T cell was co-cultured for three days with MSCs prior to assessing IFN-gamma and IL-2 production by respective Quantikine ELISAs.

### Monitoring antigen up-take and processing

To evaluate the effect of UM171a on OVA uptake, 1.5 × 10^4^ cells were seeded per well in a 24 well plate. On the following day, cells were treated with DMSO or UM171a (1000 nM) for three days. Once the UM171 treatment period was completed, 1 μg/ml of Alexa Fluor® 647-conjugated OVA was added on cells for 2 h prior to their trypsinization and washing with cold PBS containing 2% FBS. Fluorescence was then assessed by flow-cytometry. For OVA processing, UM171a- or DMSO-treated MSCs (as explained above) were incubated with 10 μg/mL OVA-DQ at 37 °C. One hour later, cells were washed, and regular media added (pulse and chase). After 3 h, cells were collected and washed with cold PBS containing 2% FBS. Fluorescence was monitored by flow-cytometry.

### Luminex analysis and IDO-1 quantification

To screen and quantify cytokine production, UM171a- or DMSO-treated MSCs were cultured for three days in the absence of serum. Once the incubation period completed, supernatants were collected, centrifuged for 10 min at 750 × g to remove any floating cells or cell debris prior to concentrating the collected media using the Amicon Ultra-4 centrifugal filters (3000 NMWL) for 45 min at 4 °C. Collected concentrate was then frozen at – 80 °C until shipped to EveTechnologies (Calgary, AB, CANADA) for luminex assessment. A similar approach was used to quantify IDO-1 production by ELISA. MSCs treated with 10 ng/ml of IFN-gamma overnight were used as positive control.

### Bioinformatics analysis

Bulk RNA seq data were downloaded from GEO (GSE138487). Gene-level count data were imported and processed in DESeq2 [17]. Expression data from OCI-AML5 cells treated with UM171a over 72 h were contrasted with data from DMSO-treated cells. The resulting differential analysis (DEG) generated a ranked list of genes using the Wald statistic, which was subsequently used for Gene set enrichment analyses [18]. The Biological process GO annotations were selected to identify gene sets up- or down-regulated by UM171a. Heatmaps were plotted in R statistical programming (using heatmap.2 function in gplots package).

### Immunization and tumor challenge studies

For therapeutic vaccination, female C57BL/6 mice (*n* = 10/group) were first subcutaneously (SC) implanted with 5 × 10^5^ EG.7 cells at day 0. Four to five days later (e.g., following appearance of palpable tumors ~ 20–40 mm^3^), mice were SC-injected with 2.5 × 10^5^ UM171a- or DMSO-treated MSCs pulsed with 5 mg/ml OVA protein for 5–6 h (detached using Accutase®). Two injections were given one week apart. Control animals received 5 × 10^5^ tumor cells alone. Vaccinated animals were followed thereafter for tumor growth.

### Statistical analysis

*P *values were calculated using the one-way analysis of variance (ANOVA), except for Fig. 3B where the student *t*-test was used. Results are represented as average mean with standard deviation (S.D.) error bars, and statistical significance is represented with asterisks: *P  < 0.05, **P < 0.01, ***P < 0.001.

## Results

### UM171a is well tolerated by primary MSCs and triggers MHCI up-regulation

**Fig. 1:**
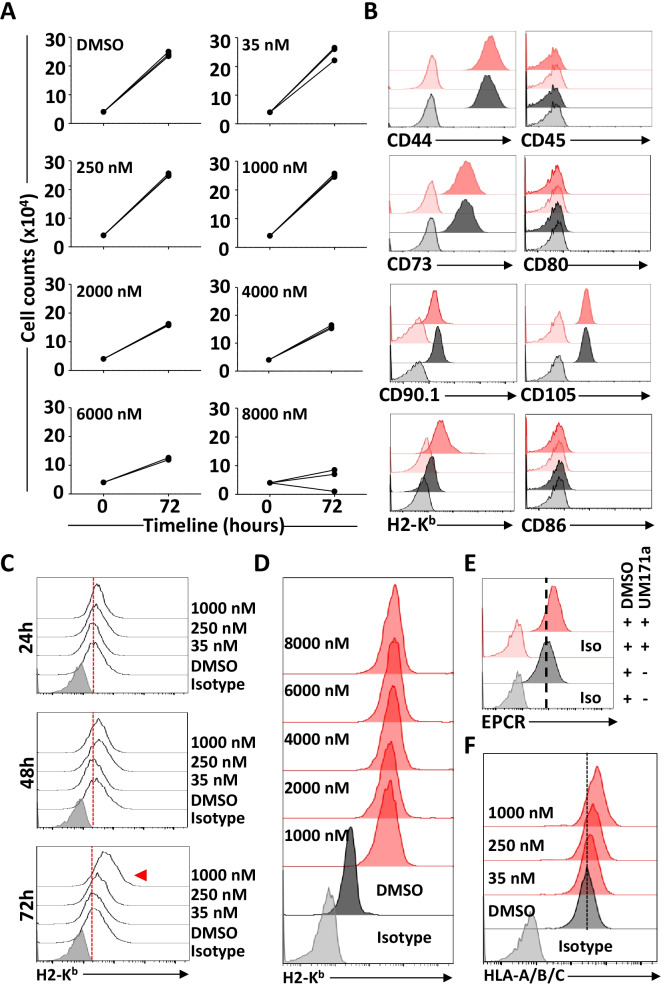
Characterizing the pharmacological effect of UM171a on MSCs. **A** Assessment of various UM171a doses on the proliferation of MSCs over a 72 h period. For this panel, *n* = 3/group. **B** Phenotypic analysis of MSCs treated with 1000 nM UM171a for 72 h. The dashed lines represent isotype signals. The isotype controls for DMSO and UM171a are shown in light gray and red, respectively. The isotype and marker staining for DMSO are shown in light and dark gray, respectively. The isotype and msrker staining for UM171a are shown in light and dark red, respectively. **C** Timeline comparison of the effect of three UM171a doses on H2-K^b^ induction. **D** Testing the effect of UM171a on H2-K^b^ using doses higher than 1000 nM. **E** Flow analysis of EPCR cell surface expression on MSCs treated with 1000 nM UM171a for 72 h. **F** Representative flow-cytometry analysis of HLA-A/B/C on the surface of human UC-derived MSCs treated with UM171a at 35, 250 and 1000 nM. All experiments were repeated at least three times

The parent UM171a compound was initially discovered by a high-throughput screening assay designed for the identification of compounds capable of triggering leukemic stem cell proliferation [19]. A series of chemical modifications were then conducted to create the final UM171a product, which effectively promotes ex vivo expansion of long-term (LT)-hematopoietic stem cells (HSCs) [20]. When further studied to decipher its potential mode of action on human CD34^+^ HSCs, UM171a was found to trigger a marked increase in the expression of several immune-related genes including human leukocyte antigens (HLA)-A and B—ortholog of the murine MHCI (aka H2-K/H2-D), beta 2-microglobulin (β2M) as well as the co-stimulatory molecule CD86 [21]. Since these specific genes are central to antigen presentation, we posited that treatment of primary murine MSCs with UM171a would trigger or enhance the expression of these genes resulting in the acquisition of antigen presentation properties. Prior to testing this hypothesis, we first identified the working concentration of UM171a by conducting MTD experiments on murine MSCs over three consecutive days. We elected to work with a UM171a concentration of 1000 nM as higher doses impair cell proliferation (Fig. 1A). Further characterizations revealed that UM171a treatment did not alter the innate MSC phenotype as the cells remained CD45 negative while expressing CD44, CD73, CD90.1, and CD105 (Fig. 1B; Additional file 1: Fig S1). Although no increase in cell surface expression of the co-stimulatory molecules CD86 nor its homolog CD80 was detected on murine MSCs (Fig. 1B), a sharp increase in H2-K^b^ expression was observed (Fig. 1B). To see if this H2-K^b^ increase requires a 72 h treatment and/or a dose as high as 1000 nM, we evaluated the effects of multiple UM171a doses (35, 250 or 1000 nM) in a timely manner. Indeed, H2-K^b^ levels were only enhanced following a three-day treatment with 1000 nM of UM171a (Fig. 1C) and remained steady up to a dose of 8000 nM (Fig. 1D). Interestingly, assessment of EPCR expression, a marker of UM171a-induced activation, followed an expression profile kinetic akin to H2-K^b^ (Fig. 1E) indicating a direct correlation between H2-K^b^ increase and enhanced EPCR expression. To ensure that these observations can be replicated using human cells, human UC-derived MSCs were treated with 1000 nM UM171a and showed a similar increase pattern in HLA-A/B/C expression (Fig. 1F). Altogether, these results indicate that UM171a is well tolerated by MSCs and can trigger a potent increase in MHCI/HLA cell surface expression.

### Treatment of murine MSCs with UM171a instills antigen cross-presentation abilities with no protagonist effect on antigen uptake and processing

**Fig. 2:**
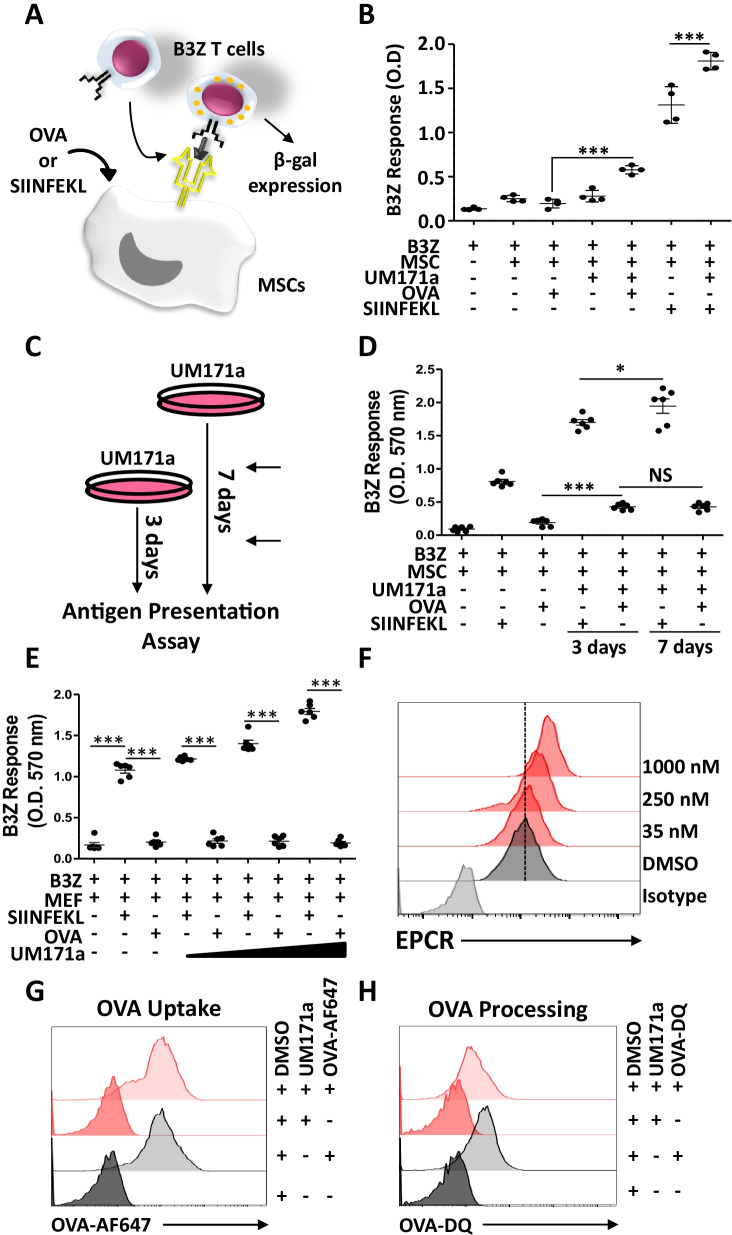
UM171a-treated MSCs cross-present soluble antigens. **A** Schematic diagram showing the design of the antigen cross-/presentation assay. **B** UM171a triggers de novo cross-presentation by MSCs and enhances antigen presentation. **C** Schematic diagram showing the design of the antigen cross-presentation assay in response to 3- or 7-day treatment. **D** OVA cross-presentation response following a 3- or 7-day treatment. **E** Antigen presentation assay conducted on primary MEFs treated with UM171a. **F** Assessment of EPCR expression by flow-cytometry on the surface of UM171a-treated MEFs. The dashed line represents the DMSO signal. **G** Flow-cytometry assessment of fluorescent OVA uptake by UM171a-treated MSCs. DMSO-treated cells are shown by gray histograms, whereas UM171a-treated cells are depicted in red. **H** Evaluating OVA processing as in panel G. All experiments were repeated at least three times. For panels B, D and E, *n* = 6/group with *P < 0.05 and ***P < 0.001

The observed increase in MHCI/HLA levels on the surface of UM171a-treated MSCs suggests that these cells may exhibit enhanced antigen presentation or the capacity to cross-present captured soluble antigens to responding CD8 T cells. We thus tested whether the identified dosing and treatment regimen affects antigen cross-/presentation by MSCs following soluble OVA protein or SIINFEKL peptide pulsing, respectively (Fig. 2A). Besides exhibiting enhanced antigen presentation (as shown by the SIINFEKL response), UM171a-treated MSCs were also able to cross-present the immunogenic OVA-derived SIINFEKL peptide (Fig. 2B) with a comparable T-cell response following longer (7 instead of 3 days) treatment regimen (Fig. 2C, D). When tested on MEFs—another non-hematopoietic cell—UM171a treatment failed to trigger antigen cross-presentation despite improved antigen presentation (Fig. 1E) and increased EPCR expression (Fig. 1F). Since the observed antigen cross-presentation effect mediated by UM171a can be potentially enhanced by increased extracellular antigen capturing and/or intracellular processing, MSCs were first treated with UM171a for three days and then, pulsed with either fluorescent OVA-AF647 (to assess antigen capturing) or OVA-DQ (to evaluate OVA processing). Compared to DMSO-treated MSCs, no increase in antigen uptake (Fig. 2G) nor antigen processing (Fig. 2H) was observed. The sum of these observations stipulates that UM171a can trigger de novo antigen cross-presentation by MSCs in a mechanism(s) independent of enhanced antigen uptake or processing.

### Reactive oxygen species (ROS) production drives antigen cross-presentation in UM171a-treated MSCs

**Fig. 3:**
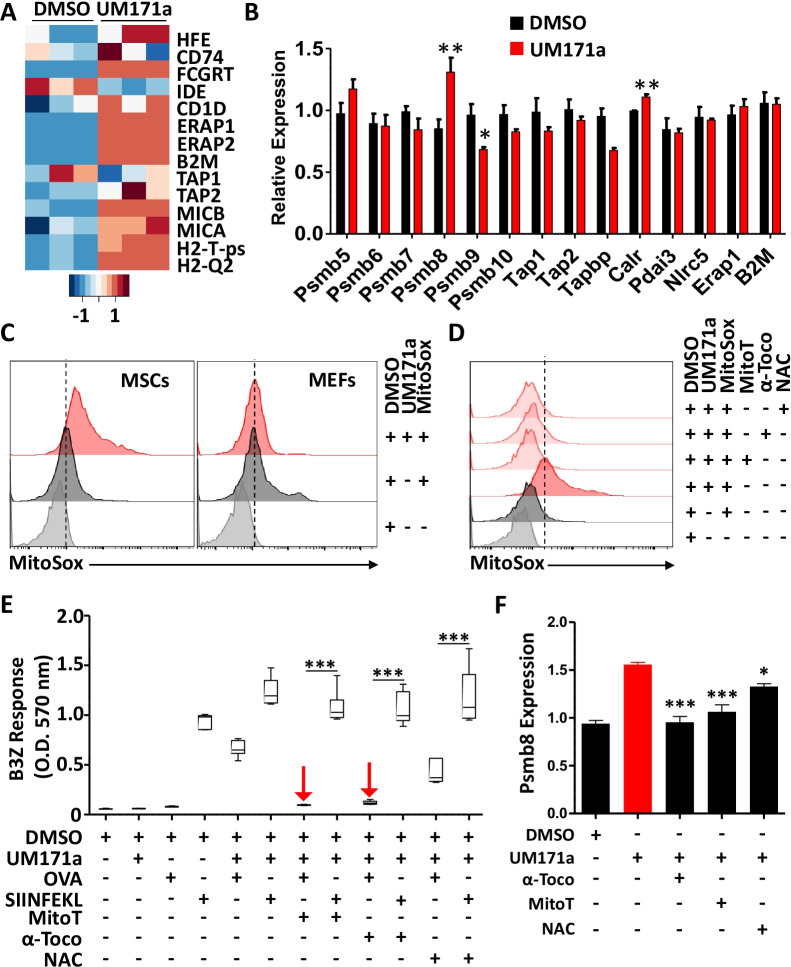
UM171a treatment leads to ROS production. **A** A heatmap showing differentially expressed genes in GO:0019883 (Antigen processing and presentation). This process is substantially up-regulated in UM171a 72 h treated group [normalized enrichment score = 2.1; FDR < 0.01]. **B** Transcript quantification of genes involved in the ERAD pathway. **C** Representative MitoSOX staining of MSCs or MEFs treated with UM171a. The dashed line represents the signal for the DMSO/MitoSOX condition. **D** Representative experiment of MitoSOX staining of UM171a-treated MSCs following antioxidant treatment. The dashed line represents the UM171a MitoSOX signal. **E** Antigen cross-presentation assay using the antioxidants MitoTEMPO (10 μM), *α*-tocopherol (800 μM), and NAC (5 mM) added at day 0 with UM171a for 72 h. Red arrows highlight the inhibitory effect of the antioxidants on antigen cross-presentation. **F** Quantification of *Psmb8* transcript in UM171a-treated MSCs undergoing co-treatment with antioxidants over 72 h. The UM171a group (positive control) is displayed in red. For panels B, E and F, *n* = 6/group with *P < 0.05, **P < 0.01 and ***P < 0.001

Since antigen up-take and processing could not explain the induced cross-presentation ability of UM171a-treated MSCs, we next wondered whether such treatment affects the endoplasmic-reticulum (ER)-associated protein degradation (ERAD) machinery. ERAD is a cellular pathway responsible for targeting misfolded proteins for ubiquitination and subsequent degradation by the proteasomal complex [22]. Analysis of publicly available transcriptomic data conducted on human HSCs revealed UM171a-mediated changes in the expression of several ERAD-related genes such as *Erap1/2*, *β2m*, *Tap1/2* as well as *H2-t* and *H2-q* molecules (Fig. 3A). Although expression of murine homolog of these genes remained steady in UM171a-treated murine MSCs, a noticeable increase in the expression of other tested genes, *Psmb8* and *Calr*, was observed (Fig. 3B). This is a salient observation for three main reasons. First, *Psmb8*—the *β*5i-subunit of the immunoproteasome—possesses a strong chymotryptic- and tryptic-like processing activity capable of generating 8–9 amino-acid peptide fragments that can efficiently fit within MHCI grooves [23, 24]. Second, *Calr* plays an important role in capturing misfolded proteins preventing their migration from ER to the golgi apparatus [25]. Third, the expression of these two genes can be induced in response to misfolded proteins that accumulate intracellularly due to aggregations or damages inflicted by elevated ROS production [26–29]. This is in line with the previous observation that treatment of human CD34^+^HSCs with UM171a induces detoxification pathways as a defense mechanism to counteract the toxic effects mediated by elevated ROS levels [21]. When we investigated UM171a-triggered ROS (more specifically superoxide anion) production in both murine MSCs and MEFs following a 72 h treatment condition, a signal was only detected in MSCs (Fig. 3C). Production of superoxide anion production was, however, completely abolished in MSCs following MitoTEMPO (an inhibitor of mitochondrial-derived ROS), vitamin E derivative *α*-tocopherol (inhibitor of lipid peroxidation), or NAC (a general antioxidant and cysteine donor) co-treatments (Fig. 3D). These observations prompted us to further explore whether ROS production predisposes MSCs to acquire antigen cross-presentation abilities. We thus co-treated UM171a-pulsed MSCs with the same anti-oxidants listed above prior to conducting an antigen presentation assay. As shown in Fig. 3E, addition of MitoTEMPO or *α*-tocopherol completely blunted antigen cross-presentation by UM171a-treated MSCs, whereas significant inhibition was observed with the use of NAC. Antigen presentation (e.g., SIINFEKL pulsing), on the other hand, remained unchanged between anti-oxidants and control treatments. To further re-enforce this hypothesis, we next compared the transcript levels of *Psmb8* in UM171a-treated MSCs co-treated with anti-oxidants. As expected, *Psmb8* expression was impaired in response to *α*-tocopherol, MitoTEMPO or NAC (Fig. 3F), clearly indicating a central role played by ROS in mediating antigen cross-presentation via de novo expression of *Psmb8*.

**Fig. 4:**
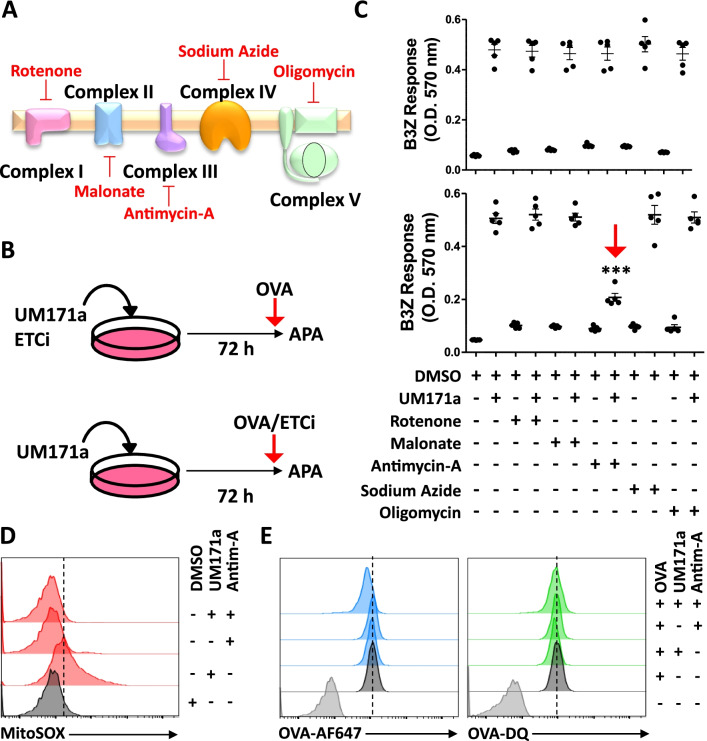
UM171a-triggered cross-presentation requires ROS production. **A** Representative diagram displaying the ETC complexes and their respective inhibitors. **B** Schematic diagram representing the experimental design of ETC inhibitor use along with UM171a. **C** Antigen presentation assay using ETCi co-treated with UM171a (upper panel) or added at day 3 during OVA pulsing. **D** Representative flow cytometry analysis of MitoSOX in MSCs co-treated with UM171a and Antimycin-A. The ETCi was added during the OVA pulsing period. The dashed line represents basal ROS levels before treatments. The dashed line represents the UM171a-induced MitoSOX signal. **E** Representative flow cytometry analysis of OVA uptake (left panel) versus OVA processing (right panel) in the absence or presence of Antimycin-A co-treatment. All experiments were repeated at least three times. The dashed lines represent the signal in the OVA-treated groups only. For panels in C, *n* = 6/group with *P < 0.05 and ***P < 0.001

ROS are generally produced by mitochondria during the process of oxidative phosphorylation [30]. More specifically, electron transfer between complexes of the electron transport chain (ETC) leads to partial reduction in oxygen to form superoxide anion [31]. Since UM171a triggers both ROS and their cognate detoxification mechanisms, it is logical to stipulate that it may act either directly or indirectly onto ETC complex(es). We thus tested the effect of various ETC inhibitors (ETCi—Fig. 4A) on MSC-mediated antigen cross-presentation as a co-treatment strategy with UM171a (e.g., since day 1) or during the antigen pulsing step (after the three-day treatment period with UM171a—Fig. 4B). Surprisingly, antigen cross-presentation by UM171a-treated MSCs was unaffected by ETCi during the co-treatment regimen (Fig. 4C—upper panel), whereas a significant decrease in B3Z activation was only observed when Antimycin-A (inhibitor of complex III) was co-treated with OVA (Fig. 4C—lower panel). Interestingly, UM171a-pulsed MSCs co-treated with Antimycin-A showed very low or absent superoxide anion production (Fig. 4D) with the absence of major effects on OVA uptake or processing (Fig. 4E). These results clearly indicate that mitochondrial-derived ROS production is the main factor driving antigen cross-presentation by UM171a-treated MSCs.

### UM171a treatment does not induce PD-L1 expression on MSCs

**Fig. 5:**
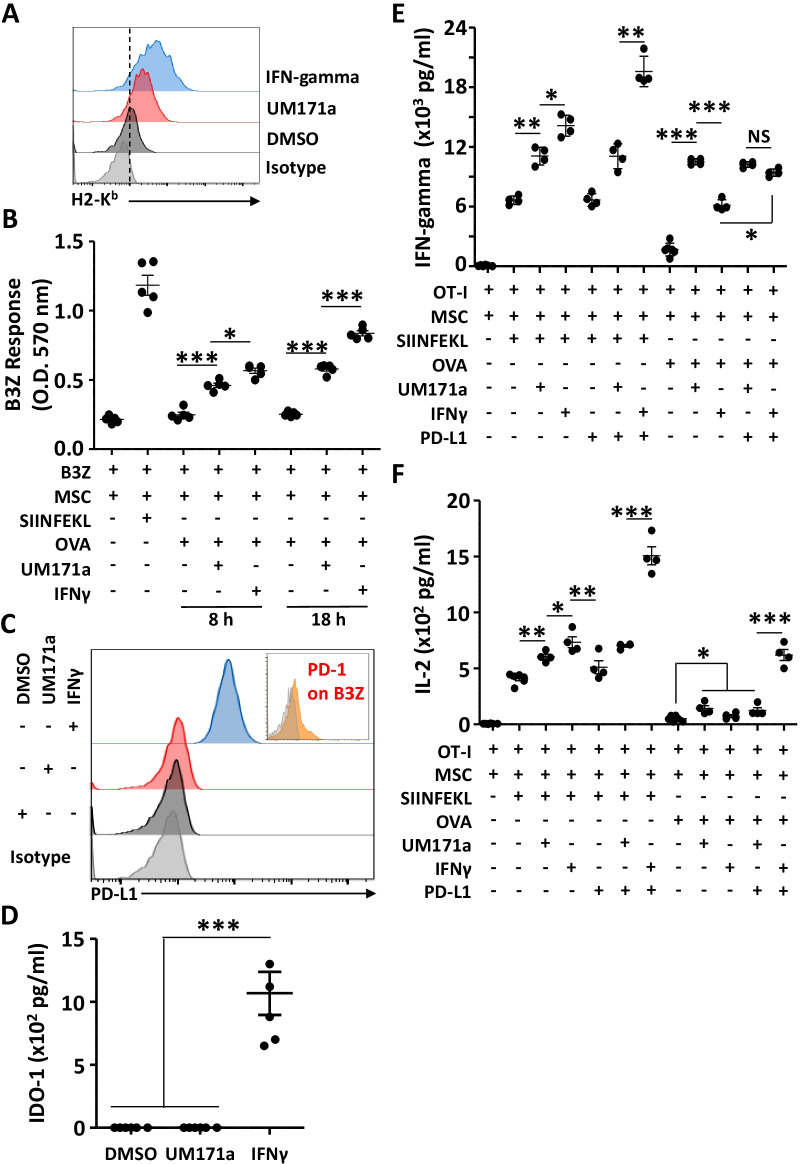
UM171a instills antigen cross-presentation properties without PD-L1 induction on the surface of MSCs. **A** Representative flow-cytometry analysis of H2-K^b^ on MSCs treated with IFN-gamma or UM171a. The dashed line represents the basal expression level of H2-K^b^ before treatments. **B** An antigen cross-presentation experiment comparing MSCs treated with UM171a versus IFN-gamma. OVA pulsing was conducting for both 8 or 18 h. **C** Representative flow-cytometry experiment assessing the expression of PD-L1 on both UM171a- or IFN-gamma-treated MSCs. The small integrated histogram represents PD-1 expression (in orange) on the B3Z cell line. **D** IDO-1 quantification by MSCs treated for 72 h with DMSO or UM171a versus 12 h with IFN-gamma (10 ng/ml). **E**, **F** IFN-gamma and IL-2 quantification by OT-I CD8 T cells in response to UM171a- or IFN-gamma-treated MSCs. The PD-L1 neutralizing antibodies were used at 0.5 μg/ml. For panels B, D, E, and F, *n* = 5/group with *P < 0.05 and ***P < 0.001

We know so far that MSC treatment with both UM171a or IFN-gamma leads to enhanced MHCI expression (Fig. 5A) and promotes antigen cross-presentation (Fig. 2B) [6]. We thus decided to compare the functional potency of both treatments in an antigen presentation assay. Since the OVA pulsing protocols for UM171a- and IFN-gamma-treatment are different (8 versus 18 h, respectively), we tested both conditions and noted a significantly higher T-cell response with the IFN-gamma treatment (Fig. 5B) most likely owing to the elevated H2-K^b^ levels following IFN-gamma treatment (Fig. 5A). Interestingly, however, UM171a did not induce de novo expression of PD-L1 on the surface of MSCs (Fig. 5C) nor IDO-1 secretion compared to IFN-gamma treatment (Fig. 5D). Since the B3Z cell line may not be highly responsive/sensitive to PD-1/PD-L1 interaction due to its low/absent PD-1 expression profile (small panel in Fig. 5C), we repeated the antigen presentation assay using primary OT-I-derived CD8 T cells and assessed their responsiveness by quantifying both IFN-gamma and IL-2 production. Although the T-cell response to SIINFEKL presentation by IFN-gamma-treated MSCs was substantially higher compared to the UM171a-treated group (Fig. 5E, F), the antigen cross-presentation ability of UM171a-treated MSCs was superior to IFN-gamma treatment (Fig. 5E, F), but became comparable to the IFN-gamma group in the presence of PD-L1 neutralizing antibodies (Fig. 5E, F). These results clearly highlight a therapeutic advantage for the use of UM171a as it precludes the negative role played by PD-L1 expression normally induced in response to IFN-gamma stimulation.

### Therapeutic vaccination using UM171a-treated MSCs delays tumor growth

**Fig. 6:**
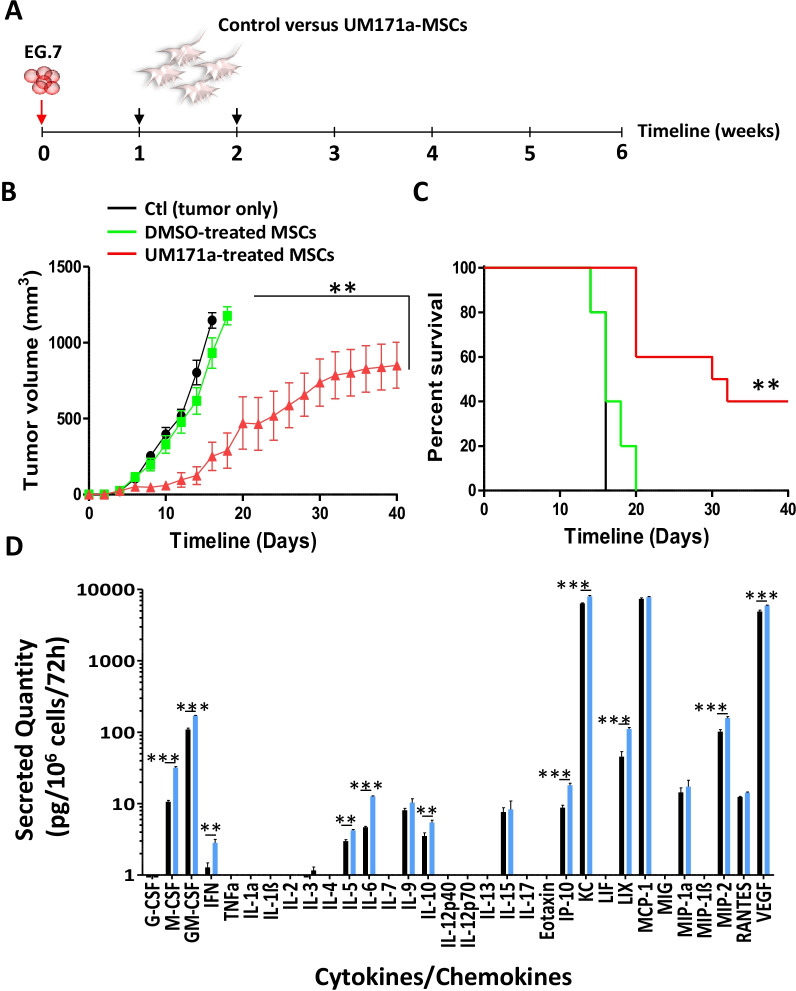
Anti-tumoral response induced by therapeutic vaccination using UM171a-treated MSCs. **A** Schematic diagram showing the experimental design used for therapeutic vaccination. **B** Assessment of tumor growth overtime following administration of DMSO-treated MSCs (green) or UM171a-treated MSCs (red) pulsed with OVA. Mice with injected EG.7 tumors are depicted in black. **C** Kaplan–Meier survival curve of the experiment shown in panel B. **D** Secretome profiling conducted on DMSO- (black) versus UM171a-treated MSCs (blue) cultured for 72 h. For this panel, *n* = 6/group with ***P < 0.001

Given the potent in vitro cross-presentation ability of UM171a-treated MSCs, we finally assessed the ability of these cells to trigger anti-tumoral immune response in immunocompetent animals with pre-established EG.7T-cell lymphomas (Fig. 6A). The SC delivery of OVA-pulsed MSCs treated with UM171a significantly delayed tumor growth compared to OVA-pulsed MSCs or untreated control mice (Fig. 6B) with a 40% survival rate reached up to 40 days post-tumor transplantation (Fig. 6C). Although this therapeutic effect can be explained by the immunogenic potential of the vaccine (e.g., OVA-derived peptides), MSCs can further modulate immunity via their capacity to secrete various immune soluble mediators [32, 33]. We thus evaluated whether UM171a affects the secretome of MSCs, hence amplifying their anti-tumoral properties. Indeed, a three-day treatment with UM171a led to significant increases in various pro-inflammatory cytokines (M-CSF, GM-CSF, IL-6, and IP-10) and chemokines (KC, LIX, and MIP-2—Fig. 6D), which are all known for their ability to recruit and modulate the activity of host-derived innate and adaptive immune cells. Altogether, these findings indicate that UM171a-treated MSCs can be effectively exploited in the design of cellular vaccines capable of triggering potent anti-tumoral responses.

## Discussion

The idea of testing the effect of UM171a on antigen presentation by culture-adapted primary MSCs stems from the initial observation that UM171a-treated human LT-HSCs up-regulate several immune-related genes such as CD86 [21]. Although de novo expression of this costimulatory molecule was undetected on the surface of treated MSCs, the expression levels of MHCI were significantly increased following UM171a treatment and correlated directly with enhanced antigen presentation. Interestingly, UM171a-treated MSCs also acquired the ability to cross-present peptides from soluble antigens without exhibiting enhanced uptake or intracellular processing of captured proteins. Instead, the cross-presentation ability of UM171a-treated MSCs requires mitochondrial-mediated ROS production. Although ROS play important physiological roles in eukaryotic cells, they are also known for their ability to disrupt proteostasis by causing protein damages and aggregation resulting in ER stress [21]. This can explain the induced de novo expression of *Psmb8* in response to UM171a treatment as a means to initiate protein processing/degradation in order to "clean-up" the cell and re-establish protein homeostasis. Under such context, we can presume that any exogenous protein (example OVA) captured by UM171a-treated MSCs is subjected to ROS-mediated oxidations/damages, which ends-up targeting the protein for degradation by *β*5i-containing proteasomes consequently resulting in the generation of stable and immunogenic peptides (Graphical abstract).

The most salient observation in this study is the direct link between UM171a-mediated ROS production and antigen cross-presentation. This is supported by the blunting effect of MitoTempo and *α*-tocopherol treatments as they directly neutralized mitochondrial-derived ROS and lipid peroxidation, respectively, impairing MSCs’ ability to activate responding T cells. Their neutralizing effect did not, however, inhibit antigen presentation as reflected by the sustained SIINFEKL stimulation, suggesting another mechanism at play for MHCI enhanced up-regulation by UM171a. This is not surprising for two reasons. First, UM171a was suggested to share a common molecular pathway with tranylcypromine and potentially other LSD1 inhibitors, which can regulate the expression of both stem cell as well as classical and non-classical MHCI-related genes [34, 35]. This may explain the functional discrepancy observed between MEF and MSC responses. More specifically, the inability of UM171-treated MEFs to cross-present can be due to the absence of ROS induction, whereas their enhanced antigen presentation can be possibly caused by UM171a-mediated epigenetic effect(s). Second, our data allude to an important role for ETC complex III in this context as co-treatment of UM171a-treated MSCs with Antimycin-A, but not other ETCi (e.g., rotenone, malonate, oligomycin, and sodium azide), during OVA pulsing impaired antigen cross-presentation. It is not clear so far if UM171a directly binds complex III or supports an indirect function associated with: i) oxidative phosphorylation, ii) TCA cycle activity, iii) regulating the expression of genes associated with complex III, or even iv) inducing hypoxia. However, complex III is the only ETC component capable of releasing superoxide anion to both sides of the inner mitochondrial membrane (e.g., matrix and cytoplasm) [36]. Although the exact Antimycin-A mode of action remains ill-defined, this ETCi was proposed to regulate ROS flow away from the mitochondrial matrix into the cytoplasm [36]. This suggests that matrix-resident ROS are central to UM171a-related cross-presentation activity as their diminished production in response to Antimycin-A impairs T-cell activation. Further studies are therefore warranted to decipher the exact UM171a mode of action alone or in concert with Antimycin-A in order to understand the implication of this molecule at the mitochondrial level.

## Conclusion

The use of IFN-gamma to promote antigen cross-presentation by MSCs highlights the pleotropic function that can be mediated by this non-hematopoietic cell following its pharmacological stimulation. However, the IFN-gamma stimulation approach is hampered by several factors including the use of an appropriate dosing, the long-term negative effect of IFN-gamma stimulation, which cannot be controlled once the cells are administered in vivo, as well as T-cell inhibition via de novo expression of the PD-L1 immune-checkpoint. UM171a has the advantage of bypassing most of these limitations as it does not seem to be negatively modulated once a pharmacological effect has taken place while triggering a pro-inflammatory phenotype without inducing PD-L1. The sum of these attributes explains the remarkable effect observed on tumor growth following therapeutic vaccination. That been said, additional studies addressing the translational potential of UM171a using human-derived MSCs are needed. Such studies may, however, face some hurdles for two main reasons. First, it is difficult to test the immunogenicity of UM171a-treated human MSCs as humanized animal models with fully reconstituted immune systems containing a wide repertoire of functional T cells remain unavailable. Second, treatment of human MSCs with UM171a can lead to different outcomes depending on the source of tissue/organ from which these cells are derived. Thus, a direct comparison between different sources of MSC populations is required to properly set the best conditions required to achieve efficient antigen cross-presentation. Nevertheless, the concept of using UM171a to drive ROS production as a means to trigger components of the immunoproteasome complex pave the path for the search of additional compounds that may act on this pathway for the future design of cancer cell vaccines as an alternative to the use of standard dendritic cells.

## Supplementary Information

**Additional file 1: Fig. S1**. Representative gating strategy used for MSC phenotypic analysis. **A**, **B** FSC-SSC gates used to detect the MSC populations prior to conduct CD45 and CD80 staining, respectively. **C**, **D** Representative dot blot for CD45 and CD80 staining, respectively.

## Data Availability

Data sets and material/reagents analyzed and/or used in this study are available upon reasonable request.

## Abbreviations

ANOVA: Analysis of variance
APC: Antigen presenting cell
β2M: Beta 2-microglobulin
Calr: Calreticulin
CPRG: Chlorophenol red-*β*-d-galactopyranoside
DC: Dendritic cell
DEG: Differentially expressed gene
EPCR: Endothelial protein C receptor
ER: Endoplasmic reticulum
ERAD: Endoplasmic-reticulum-associated protein degradation
ETC: Electron transport chain
FBS: Fetal bovine serum
GM-CSF: Granulocyte macrophage-colony stimulating factor
IL: Interleukin
IFN: Interferon
IP-10: Interferon gamma-induced protein 10
KC: Keratinocytes-derived chemokine
LPS: Lipopolysaccharide
LT-HSC: Long term-hematopoietic stem cells
LIX: Lipopolysaccharide-induced CXC chemokine
M-CSF: Macrophage-colony stimulating factor
MEF: Murine embryonic fibroblast
MHCI: Major histocompatibility complex I
MIP-2: Macrophage inflammatory protein-2
MSC: Mesenchymal stromal cell
MTD: Maximum tolerated dose
NAC: *N*-Acetylcysteine
NRF2: Nuclear factor erythroid 2–related factor 2
OVA: Ovalbumin
PSMB8: Proteasome 20S subunit beta 8
ROS: Reactive oxygen species
SC: Sub-cutaneous
SD: Standard deviation
UC: Umbilical cord
VEGF: Vascular endothelial growth factor
