# Supplementary figures and images for: UM171A-induced ROS promote antigen cross-presentation of immunogenic peptides by bone marrow-derived mesenchymal stromal cells
Source: Stem Cell Res Ther. 2022 Jan 10;13:16. doi: 10.1186/s13287-021-02693-z (PMC8751335; doi:10.1186/s13287-021-02693-z)

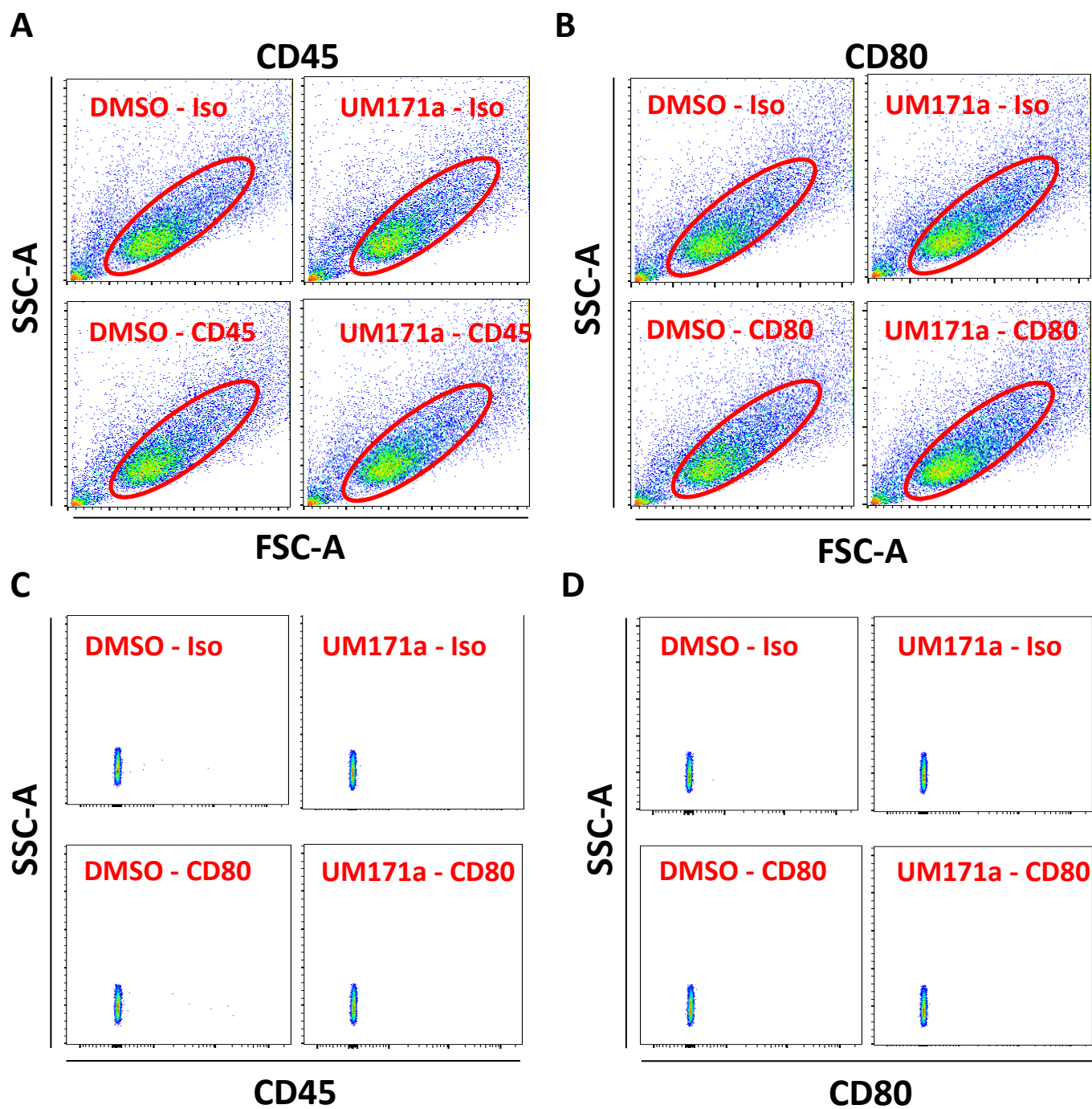

Supplement: Supplementary file 1 — Additional file 1: Fig. S1. Representative gating strategy used for MSC phenotypic analysis. A, B FSC-SSC gates used to detect the MSC populations prior to conduct CD45 and CD80 staining, respectively. C, D Representative dot blot for CD45 and CD80 staining, respectively. [file 13287_2021_2693_MOESM1_ESM.pdf]
